# Astragalus polysaccharide: implication for intestinal barrier, anti-inflammation, and animal production

**DOI:** 10.3389/fnut.2024.1364739

**Published:** 2024-05-02

**Authors:** Hui Liang, Siming Tao, Yanya Wang, Jing Zhao, Chang Yan, Yingjie Wu, Ning Liu, Yinghe Qin

**Affiliations:** ^1^State Key Laboratory of Animal Nutrition and Feeding, China Agricultural University, Beijing, China; ^2^Beijing Advanced Innovation Center for Food Nutrition and Human Health, China Agricultural University, Beijing, China

**Keywords:** anti-inflammation, Astragalus polysaccharide, intestinal barrier, microbiota, short-chain fatty acids

## Abstract

Intestine is responsible for nutrients absorption and plays a key role in defending against various dietary allergens, antigens, toxins, and pathogens. Accumulating evidence reported a critical role of intestine in maintaining animal and human health. Since the use of antibiotics as growth promoters in animal feed has been restricted in many countries, alternatives to antibiotics have been globally investigated, and polysaccharides are considered as environmentally friendly and promising alternatives to improve intestinal health, which has become a research hotspot due to its antibiotic substitution effect. Astragalus polysaccharide (APS), a biological macromolecule, is extracted from astragalus and has been reported to exhibit complex biological activities involved in intestinal barrier integrity maintenance, intestinal microbiota regulation, short-chain fatty acids (SCFAs) production, and immune response regulation, which are critical for intestine health. The biological activity of APS is related to its chemical structure. In this review, we outlined the source and structure of APS, highlighted recent findings on the regulation of APS on physical barrier, biochemical barrier, immunological barrier, and immune response as well as the latest progress of APS as an antibiotic substitute in animal production. We hope this review could provide scientific basis and new insights for the application of APS in nutrition, clinical medicine and health by understanding particular effects of APS on intestine health, anti-inflammation, and animal production.

## 1 Introduction

Intestine, one part of the digestive tract, is not only responsible for digestion and absorption of dietary nutrients, but also plays a key role in immune homeostasis, protecting the body from various dietary allergens, antigens, toxins, and pathogens. Nowadays, accumulating evidence reported a critical role of intestine health in animal and human health maintenance (1). Indeed, intestinal homeostasis disorder may result in impaired morphology and function of intestine, reduced digestion and absorption capacity, increased diarrhea rate, reduced feed intake, and growth retardation (2–4). Hence, intestinal health status is crucial for an optimal production result and sustainable animal production (5). With raise public attention to intestinal health, intestine health has become a hot research topic in recent years (6). Intestinal barrier (physical barrier, biochemical barrier, and immunological barrier) composed of epithelial cells, microbiota, immune cells, and their secretions is key for intestinal health and involved in protecting body from the penetration of harmful entities (e.g., microorganisms, luminal antigens, and luminal proinflammatory factors) to maintain a stable internal environment (7). Moreover, dysfunction of intestinal barrier is believed to contribute to a broad range of diseases, such as inflammatory bowel disease (IBD), colorectal cancer, chronic liver disease, type 1 diabetes, and obesity (8, 9).

Since the use of antibiotics as growth promoters in animal feed has been restricted in many countries, accumulating evidence reported that probiotics, vitamins, minerals, amino acids, or plant extracts, etc. have been used to regulate intestinal barrier. Notably, plant extracts are natural, and multi-compounds products formed through an extraction and separation process from plant and exert positive effects on the integrity of the intestinal barrier with high efficiency and no residue. Astragalus comes from a type of leguminous herb and known as Huang Qi in China. Astragalus have been widely used to replenish qi by soaking into the water in folk. Astragalus supplements contains polysaccharides, saponins, flavonoids, and etc. (10). Among these biologically active ingredients, Astragalus polysaccharide (APS), a water-soluble heteropolysaccharide extracted from the stem or dried root of astragalus, is the most abundant and important active substance in Astragalus (11). Chemical composition analysis indicates that APS is mainly composed of glucose, galactose, rhamnose, mannose, xylose, arabinose, glucuronic acid, and galacturonic acid (12). Growing pharmacological and clinical trials had shown that APS is used to protect and support the immune system (13). In addition, APS exerts anti-oxidation, anti-aging, anti-fibrosis, anti-tumor, antiviral and antibacterial, blood sugar reduction, blood lipid reduction, anti-fibrosis, and radiation protection effects (14). Studies showed that traditional Chinese medicine (TCM) have applied as effective method to modify intestinal dysfunction, regulate structure and function of gut microbiota, reduce inflammation response, as well as cell repair intestinal barrier. Likewise, emerging evidence focused on the regulation of intestinal health by APS have indicated the beneficial effects and underlying mechanisms involved in intestinal barrier maintenance, intestinal microbiota regulation, immune response, and redox homeostasis (15–17).

In this review, we firstly outlined the source and structure of APS. And then, we highlighted recent advancements on APS as a potential therapeutic intervention for intestinal disease associated with intestinal barrier dysfunction, intestinal microbiota disorder, intestinal inflammatory response, as well as intestinal oxidative stress response. We hope this review could provide scientific basis and new insights for the application of APS in nutrition, clinical medicine, and health by understanding particular effects of APS on intestine homeostasis and immune response.

## 2 Characteristics of Astragalus polysaccharide

Polysaccharides are polymers constituted of more than 10 monosaccharides with condensation reaction (18, 19). Polysaccharides are widely present in plants, algae, animals and microorganisms (bacteria, fungi, and yeasts) and generally obtained from plants through extraction, separation, and purification (20). Growing evidences revealed that polysaccharides possess complex biological activities and a variety of biological functions involved in antioxidant, antitumor, antiviral, immune regulation activities and so on (21). Of note, polysaccharides are widely present in TCM and considered as one of the important bioactive ingredients in TCM (22). Additionally, polysaccharides can be divided into homopolysaccharides and heteropolysaccharides according to the composition of monosaccharides. Homopolysaccharides refer to polysaccharides composed of only one monosaccharide, while heteropolysaccharides are polysaccharides composed of two or more monosaccharides. APS, a water-soluble heteropolysaccharide, is extracted from a common Chines herbal plant (*Astragalus membranaceus*) and considered as important bioactive components of *Astragalus membranaceus* (23).

Astragalus polysaccharide process complex biological activities including anti-inflammation, antioxidant, antiviral, anticancer, and immune functions (16, 24–27) and are applied as an additive with non-toxic side effects, low cost, and no residue. Indeed, polysaccharides are macromolecules with the chemical structures (primary, secondary, tertiary, and quaternary structures) which contribute to its biological activities (28, 29). Nevertheless, polysaccharide structural analysis indicated that polysaccharide structures are very complex and comprehensive structures were characterized by monosaccharide composition, Fourier transform infrared and nuclear magnetic resonance spectroscopy (NMR) analysis (29, 30). Although APS is formed by galactose, glucose, mannose, rhamnose, xylose, arabinose, glucuronic acid, and galacturonic acid with condensation reaction (12), but content and monosaccharide compositions of APS obtained from different original materials, different areas, and different extraction methods contribute to differentiated health benefits. The biological activities of APS are related to their chemical structure. Extraction, separation, and purification are essential steps for the APS structure determination, and then structural analysis performed by high performance liquid chromatography (HPLC), NMR, and other methods (12). To date, water extraction, enzymatic hydrolysis extraction, ultrasonic wave extraction, and microwave-assisted extraction are commonly used methods in APS extraction (31). A previous study reported that APS extracted by hot water was performed NMR analysis, structural analysis suggested that APS is a kind of glucan, the main chain is connected by α-1,4-glycoside bonds and the branch chain is α-1,6-glycoside bonds (12, 32). Nevertheless, four APSs were obtained with an ethanol precipitation procedure. Molecular weight and monosaccharide composition analysis indicated that ASP1 with molecular weight 257.7 kDa is consisted of glucose, ASP2 with molecular weight 40.1 kDa is consisted of arabinose, ASP3 with molecular weight 15.3 kDa is consisted of rhamnose, glucose, and galactose, and ASP4 with molecular weight 3.2 kDa is consisted of galactose and arabinose (33). Furthermore, HPLC method was performed to define the monosaccharide composition of APS, results indicated that APS was composed of fucose, arabinose, galactose, glucose, and xylose with molar ratios of 0.01:0.06:0.20:1.00:0.06 (34). Simultaneously, structure of APS determined by gas chromatography (GC) and Fourier transmission-infrared spectroscopy (FT-IR) suggested that ASP was composed of arabinose, mannose, glucose, mannose, and galactose and with a ratio of 0.0992:1.26:1.00:0.015 (35). In addition, a literature reported that monosaccharide formation of APS was defined by HPLC and other methods, and analysis showed that APS-I with molecular weight 1699.1 kDa was composed of arabinose and glucose (1:3.45) and APS-II with molecular weight 1197.6 kDa was composed of rhamnose, arabinose and glucose (1:6.25:17.86) (36).

## 3 Effects of Astragalus polysaccharide on intestinal barrier

**Figure 1:**
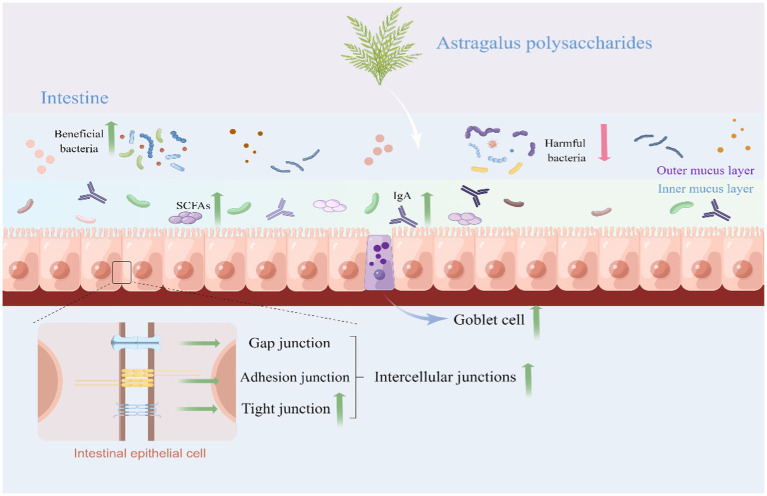
Schematic representation for effects of Astragalus polysaccharide on the intestinal health (By Figdraw). APS could protect the intestine from damage by upregulating the intestinal tight junction protein and enhancing goblet cell number. APS improve the intestinal immune response mainly by regulating sIgA secretion, cytokines production, and immune cells proliferation and differentiation. APS protects the intestinal biochemical barrier by regulating composition and structure of gut microbiota and SCFAs levels. APS, Astragalus polysaccharide; SCFAs, Short-chain fatty acids; and sIgA, Secretory immunoglobulin A.

Intestinal barrier composed of physical barrier, biochemical barrier, and immunological barrier plays a key role in preventing the passage of harmful or unwanted substances from entering the internal environment which is a crucial for humans and animals health (37–39). Dysfunction of intestinal barrier function would lead to intestinal diseases such as enteritis, IBD, celiac disease, irritable bowel syndrome (IBS), and colorectal cancer (40–42). A variety of factors, such as food antigens, pathogenic organisms, and toxins, have been reported to disorder intestinal barrier integrity, which in turn lead to a reduced animal growth performance and animal production quality (43). Schematic representation for effects of Astragalus polysaccharide on the intestinal health was presented in Figure 1.

### 3.1 Astragalus polysaccharide improves the intestinal physical barrier

The intestinal physical barrier is a single-cell layer composed of intestinal epithelial cells, intercellular junctions, and intestinal mucosa, which separates the intestinal luminal contents from the internal milieu (44, 45). Furthermore, the functionally specialized epithelial cells contain enterocytes, Paneth cells, goblet cells, tuft cells, enteroendocrine cells, and microfold cells (46, 47). Enterocytes account for >80% of epithelial cells are specialized to absorb and export luminal nutrients (48). Goblet cells secrete mucins to protect mucous membranes, enteroendocrine cells secrete peptide hormones and Paneth cells produce a number of antimicrobial peptides (49, 50). Additionally, intercellular junctional complexes including tight junctions (TJ), adherens junctions, gap junctions, and desmosomes provide contact or tightly bound between neighboring cells and play a critical role in the regulation of paracellular permeability and epithelial barrier integrity (42, 51). Notably, TJ composed of Occludin, Claudins, and Zonula occludens have been extensively studied and are responsible for intestinal barrier function (52). Accumulating studies suggested that TJ are crucial for the maintenance of epithelial barrier integrity by selectively transporting essential molecules and preventing harmful substances from entering into the internal environment (42, 53, 54).

A variety of factors, such as reactive oxygen species (ROS), infection, cytokines, and pathogens, have been reported to disorder intestinal physical barrier. Oxidative stress occurs when an imbalance emerged between the ROS production and antioxidant system, which induced intestinal cells apoptosis and disturbed abundance and distribution of TJ proteins resulting in damage of the epithelial barrier (55–57). Furthermore, ROS significantly enhanced crypt depth (CD) and reduced villus height (VH) of the intestine and ratio of villus height/crypt depth (VH/CD) (58). Emerging studies had shown that APS could inhibit inflammation, repair the integrity of intestinal barrier, and ultimately improve digestion and absorption of nutrients. Indeed, a previous study demonstrated that lipopolysaccharide (LPS)-challenged weaned piglets supplemented with 800 mg/kg APS enhanced superoxide dismutase (SOD) and total antioxidant capacity (T-AOC) in serum, and enhanced abundance of claudin and occluding in the jejunum (44). Similarly, BALB/c mice exposed to *Salmonella typhimurium* (*S. t.*). supplemented with 200 mg/kg APS improved intestinal barrier through enhancing mRNA expression of ZO-1, Occludin, and Claudin-1 in jejunum and attenuating inflammation response (59). Furthermore, immunosuppressed broilers supplemented with 900 mg/kg γ-irradiation APS alleviated cyclophosphamide (CPM)-induced intestinal mucosa damage and increased jejunal goblet cell number (60). Collectively, these studies have shown that APS could reduce the epithelial cells loss caused by inflammation and repair the integrity of intestinal barrier.

### 3.2 Astragalus polysaccharide improves the intestinal biochemical barrier

Intestinal microbial barrier refers to commensal microorganisms (bacteria, archaea, fungi, and viruses) colonized in the digestive tracts and have been reported to play potential role in protecting against external stimuli, modulating immunity, modulating the metabolism of lipids and bile acids as well as neuromodulation (38, 61–63). In general, gut bacteria mostly account for gut microorganisms and members of Firmicutes, Bacteroidetes, Actinobacteria, Proteobacteria, Fusobacteria, and Verrucomicrobia have been identified as major bacterial phylum of gut microbiota (64, 65). Mounting studies suggested that intestinal microbiota maintain a symbiotic relationship with the host (66) and play a critical role in animal health maintenance and the pathogenesis of intestinal diseases, such as IBS, IBD, celiac disease, and colorectal cancer (67). Growing evidence demonstrated that gut microbiota not only provide essential capacities for the metabolism of nutrients, but also participate in regulating the integrity and function of the intestinal barrier in a homeostatic balance (68, 69). Therefore, gut microbiota homeostasis is vital for intestinal barrier integrity and the underlying mechanism are largely unknown (70–72). Gut microbiota provide essential capacities for the fermentation of non-digestible substrates like dietary fibers and endogenous intestinal endogenous intestinal mucus (73). Emerging evidence indicated that APS might improve intestinal barrier through promoting colonization of good bacteria, preventing pathogenic bacteria from invasion and growth, eliminating ROS and attenuating intestinal inflammation (56, 74, 75). A previous study found that dietary diet supplemented with 220 mg/kg APS enhanced the abundance of beneficial bacteria numbers (*Lactobacilli* and *Bifidobacteria*) and reduced *E. coli* abundance in the gut (76). Similarly, broiler supplemented with 900 mg/kg γ-irradiation APS reduced abundance of *Bacteroides*, *Faecalibacterium*, and *Butyricicoccus*, enhanced the abundance of *Ruminococcaceae UCG-014*, *Negativibacillus*, *Shuttleworthia*, *Sellimonas*, and *Mollicutes RF39_norank*, and elevated butyrate concentration in cecum (77). Moreover, alcoholic fatty liver disease (AFLD) mice were given 22 mg/kg corresponding polysaccharide solution (APS with polysaccharide content of 62.73%) markedly enhanced *Bacteroides S24-7* abundance, reduced abundance of *Clostridiales* and *Lachnospiraceae*, and reduced ratio of Firmicutes to Bacteroidetes (78), and ultimately elevated the abundance of beneficial bacteria and reduced the abundance of pathogenic bacteria.

Short-chain fatty acids (SCFAs) are organic linear carboxylic acids with two to six carbon atoms and are mainly produced by the gut microbiota via the fermentation of complex carbohydrates and fibers (79). Natural polysaccharides are favorable for the production of SCFAs (80, 81). Mounting evidence indicated that SCFAs play crucial roles in maintenance of intestinal health including regulation of intestinal barrier, intestinal epithelial cell growth and function, and inflammatory response modulation (82–84). SCFAs are mainly composed of acetic acid, propionic acid, and butyric acid, the main of which is butyric acid, which consumes oxygen, creating an anaerobic condition for the intestinal tract and prevent aerobic pathogens from invasion in the gut. Correspondingly, reduced SCFAs level in gut contributed to the enhanced intestinal permeability and intestinal diseases (85). APS as a natural polysaccharide has been reported to enhance SCFAs levels and modulate composition and function of gut microbiota (17). Type II diabetes mice supplemented with 600 mg/kg APS significantly enhanced fecal SCFAs level, G-protein-coupled receptor 41/43 expression, and TJ proteins (Occudin and ZO-1) abundance (86). Furthermore, APS enhanced secretion of glucagon-like peptide-1 (GLP-1) in serum and improved intestinal microbial barrier, resulting in alleviation of diabetes symptoms in mice. Similarly, a basal diet with 800 mg/kg APS markedly enhanced the level of acetic acid, propionic acid, isobutyric acid, and butyrate in colon and enhanced the colonic microbial population and diversity (87). In addition, the literature also suggested that APS (200 mg/kg) attenuated the intestinal injury caused by necrotic enteritis in broiler through enhancing the concentrations of propionic acid, butyric acid, isobutyric acid, and hexanoic acid in the ileum (88). Collectively, APS exerts intestinal barrier protection through promoting the growth of beneficial microbiota, inhibiting colonization of pathogenic bacteria, and elevating level of SCFAs. The effects of APS on the intestinal biochemical barrier can be seen in Figure 1.

### 3.3 Astragalus polysaccharide improves the intestinal immunological barrier

Intestine is not only responsible for nutrient digestion and absorption, but is also the largest immune organ comprised around 80% immune cells in the body (89, 90). The intestinal immune barrier is mainly composed of secretory immunoglobulin A (sIgA), gut associated lymphoid tissue (GALT), cytokines and other immune-producing substances, which play a key role in fighting against pathogens or toxins (90, 91). sIgA constitutively localize in mucosal secretions and serve as the first line of defense in blocking microorganisms from attaching to, colonizing and invading epithelial cells (92). Moreover, mounting evidence demonstrated that sIgA play a crucial role in regulating TJ proteins expression, shaping commensal microbiota composition, and maintaining epithelial barrier integrity and immune homeostasis (92–94). GALT include Peyer’s patches (PPs), numerous isolated lymphoid follicles (ILF), mesenteric lymph nodes (MLN) and diffuse GALT would modulate cytokine/chemokine production and immune cell function (91, 95). Additionally, excessive cytokine production aggravates intestine inflammation and intestinal barrier impairment (96). Interestingly, accumulating evidence reported that APS exerts the potentiality to promote the activities of immune cells (e.g., macrophages, natural killer cells, dendritic cells, T lymphocytes, B lymphocytes, and microglia) and regulates the production of cytokines and chemokines (16, 97, 98). A previous study was conducted to examine the effects of APS (*in ovo* injection) on number of immune cells, sIgA, and intestinal immune-related genes expression in broiler chickens. Results found that APS injection at 2 or 4 mg *in ovo* significantly enhanced VH and VH/CD ratio, increased IgA^+^ cells population and sIgA content, and enhanced mRNA expressions of interleukin (IL)-2, IL-4, interferon gamma (IFN-γ), and Toll-like receptor (TLR)-4 (99). Similarly, *in ovo* administration of Newcastle disease vaccine (NDV) conjugated with APS exerts beneficial effects on the intestinal mucosal immunity of chicks through enhancing the levels of slgA and the abundance of IgA^+^ cells in duodenal lamina propria and villi when compared with NDV treatment alone (100). Furthermore, broilers supplemented with 300 mg/kg APS improved the intestinal mucosal immune barrier function of broilers by enhancing mRNA expression of Occludin, Claudin-1, ZO-1, and MUC2 in small intestine, and improved growth by enhancing concentration of immunoglobulins (Ig) A, IgM and IgG, and lowing concentrations of TNF-α, IL-1β, IL-6, and diamine oxidase (DAO) in serum (74). In addition, broilers administrated with 0.5, 1, or 2 mg APS in 0.5 mL saline enhanced VH/CD ratio, IgA^+^ cells population, sIgA levels when compared with vaccinated control group and one non-vaccinated negative control group (101) (Table 1).

**Table 1:** Effects of Astragalus polysaccharide on the intestinal health.

Source	Application form	Experiment object	Main function	Reference
Astragalus polysaccharide	Dietary supplementation with 800 mg/kg	Weaned piglets	Improved the protein expression of Claudin and Occludin in the jejunum	(44)
Gamma-irradiated Astragalus polysaccharide purity 87.64%	Dietary supplementation with 900 mg/kg	Arbor Acres broiler chicks	Enhanced the number of jejunal goblet cells	(60)
Astragalus polysaccharide	Dietary supplementation of 200 mg/kg	BALB/c mice	Enhanced the gene expression of ZO-1, occludin and claudin-1 in the jejunum	(59)
Sulfated *Astragalus* polysaccharide purity 97%	Injected intramuscularly with 8 mg/kg of BW	Arbor Acres broiler chicks	Elevated VH	(102)
Astragalus polysaccharides purity 80%	Dietary supplementation with 200 mg/kg	Arbor Acres broiler chicks	Reduced CD and increased jejunum VH/CD ratio	(88)
Astragalus polysaccharide	5 mL compound solution by oral administration	Neonatal piglets	Improved VH and the VH/CD ratio	(103)
Astragalus polysaccharide purity 70%	0.6 g/L to drinking water	Muscovy ducks	Improved VH and the VH/CD ratio in the small intestine	(104)
Gamma-irradiated Astragalus polysaccharide purity 87.64%	Basal diet with 600 mg/kg	Ross-308 chicks	Enhanced VH, VH/CD ratios and GCs numbers	(105)
Astragalus polysaccharide purity 91.9%	Injected with 2 or 4 mg of APS in 0.5 mL physiological saline *in ovo*	Arbor Acres broiler eggs	Boosted IFN-γ, IL-2, IL-4 gene expression, TLR-4 genes, and the sIgA levels	(99)
Astragalus polysaccharide purity 70.23%	Dietary supplementation with 300 mg/kg	Arbor Acres broilers chicks	Enhanced the levels of serum IgA and reduced the gene levels of TNF-α, IL-1β, and IL-6 and the activity of DAO	(74)
Astragalus polysaccharide	A concentrated solution (2 mg/mL) was prepared in 0.9% physiological saline	SPF Leghorn fertilized eggs	Enhanced the levels of slgA and the abundance of IgA^+^ cells	(100)
Astragalus polysaccharide purity 70%	Oral administration of 0.5 mL (1, 2, and 4 mg/mL)	Hy-Line chickens	Promoted the growth of IgA^+^ cells in jejunum and the secretion of sIgA	(101)
Astragalus polysaccharide	Dietary supplementation with 400 mg/kg	C57BL/6 J mice	Promoted the growth of beneficial bacteria *Allobaculum* and *Lactobacillus*	(56)
Astragalus polysaccharide purity 62.73%	Given 22 mg/kg corresponding solution	SPF Kunming mice	Enhanced the abundance of beneficial bacteria Bacteroides S24-7 and decreased the abundance of Clostridiales and Lachnospiraceae	(78)
Astragalus polysaccharide	Dietary supplementation with 220 mg/kg	Hy-Line chicks	Enhanced the concentrations of beneficial bacteria numbers (*Lactobacilli* and *Bifidobacteria*) and cut down the concentrations of harmful bacteria numbers (*E. coli*)	(76)
Astragalus polysaccharide purity 87.64%	Dietary supplementation with 900 mg/kg	Arbor Acres broilers chicks	Reduced the abundance of Bacteroides, Faecalibacterium, Butyricicoccus, and increased OTUs	(77)
*Astragalus membranaceus* polysaccharide	Dietary supplementation with 600 mg/kg	Db/db mice	Enhanced content of SCFAs, the expression of G-protein-coupled receptor 41/43, Occudin, and ZO-1	(86)
Astragalus polysaccharide purity 80%	Dietary supplementation with 800 mg/kg	Weaned piglets	Enhanced the levels of SCFAs	(87)

## 4 Anti-inflammatory properties of APS

Currently, APS derived from natural sources has been reported to play an important role in regulating inflammatory response (106, 107). TLRs and NoD-like receptors (NLRP) have been reported to play important role in pro-inflammatory cytokines expressions regulation (108). Emerging evidence revealed that APS could inhibit the expression of NOD-like receptor thermal protein domain associated protein 3 (NLRP3) inflammasome in colon tissue, and reduced production of IL-18 and IL-1β (109). Interestingly, a previous study revealed that APS alleviated inflammatory damage of the BPD cell model through inhibiting the activation of nuclear factor-κB (NF-κB) and reducing mRNA and protein expression levels of IL-8 and intercellular adhesion molecule 1 (ICAM-1) in bronchopulmonary dysplasia (BPD) (110). It is speculated that APS may be a safe alternative to glucocorticoid in the treatment of BPD. Furthermore, a rat model of pulmonary arterial hypertension (PAH) induced by injection of monocrotaline, which was intraperitoneally injected with APS (200 mg/kg, once every 2 days) for 2 weeks leading to reduced mRNA expression of pro-inflammatory mediators TNF-α, IL-1β, and IL-6 and inflammation alleviation (111). Correspondingly, APS could inhibit the activation of phosphorylation level of IκBα, thereby inhibiting the NF-κB signaling pathway and improving PAH induced by monocrotaline. IBD, a kind of intestinal disease, has become a global burden with rapidly increasing incidence and prevalence in both industrialized countries and developing countries (112–114). Although the etiology of IBD is still unknown, previous reports had shown that the imbalanced production of pro-inflammatory cytokines and anti-inflammatory cytokines contributed to intestinal tissue damage (115). Intriguingly, dextran sulfate sodium (DSS)-induced mice colitis daily intraperitoneal injection with 0.5 mL of APS (200 mg/kg) for 3 days remarkedly reduced phosphorylation level of NF-κB and downregulated the mRNA expression of TNF-α, IL-1β, IL-6, and IL-17 (116). These results demonstrated that APS function as a natural active ingredient to treat ulcerative colitis. In a LPS-challenged Caco-2 cells model, APS addition (100 or 200 μg/mL) in cell culture remarkedly downregulated mRNA expression of TNF-α, IL-1β, and IL-8 in a dose manner (117). Consequently, result indicated that APS exerts anti-inflammatory properties on LPS-infected Caco-2 cells and is regarded as a preventive therapy for LPS induced intestinal cells damage. In addition to APS, honey-processed APS (HAPS) is a product that Radix Astragalus mixed with honey, which exhibits better efficacy pharmacological activity (118). Correspondingly, HAPS alleviated LPS-induced inflammatory responses in RAW264.7 cells by significantly reducing NO concentration and the expression of TNF-α, IFN-γ, IL-1β, and IL-22. These results indicated that anti-inflammatory activities of HAPS were more effective than those of APS (119). Furthermore, LPS-induced inflammatory lung injury mice orally administrated with 200 mg/kg APS for 14 consecutive days significantly reduced neutrophilic infiltration, phosphorylated NF-κB expression level and relative expressions of ICAM-1, Il-1β, Il-6, and TNF-α (120). Taken together, APS functions as an anti-inflammatory agent in animals and exerts its anti-inflammation mainly by inhibiting NF-κB signaling pathways and reducing expression of pro-inflammatory cytokines.

## 5 Effects of APS on animal production

The efficiency of animal production is closely related with the economic benefits of animal husbandry (121). Nowadays, the general use of antibiotics in animal feed is banned, alternatives to antibiotics are urgently needed in animal agriculture (122). Emerging evidence demonstrated that some polysaccharides could function as antibiotics alternatives to inhibit pathogens colonization and promote animal growth performance (123, 124). APS is a kind of polysaccharides and has been investigated for its effects on animal growth performance (74). A study reported that APS enhanced average daily gain (ADG) and feed conversion rate (FCR) through improving VH and VH/CD ratio, reducing immunological stress, as well as enhancing the intestinal barrier function in LPS-challenged piglets (125). Furthermore, 0.1% APS supplementation improved ADG and F/G ratio, enhanced apparent ileal digestibility (AID), and the contents of most essential amino acids and non-essential amino acids in the serum in early-weaned piglets (126). In addition, *in ovo* injection of 2 mg APS per egg significantly enhanced VH and the ratio of VH/CD and improved intestinal morphology and development of chicks, resulting in marked increase on average daily feed intake (ADFI), body weight (BW), and FCR of layer chicks (127). Interestingly, recent studies revealed that APS could regulate lipid metabolism and adipogenesis (128). A previous study reported that *in ovo* injection of 4.5 mg APS enhanced carcass percentage, reduced abdominal fat, as well as reduced educed triglycerides, total cholesterol, low-density lipoproteins, and very low-density lipoproteins in the plasma of broilers (129). When compared with control group, APS treatment improved meat quality and feed conversion rate by reducing fat metabolism. This result is attributed to induced expression of amylase by restraining the activity of other intestinal digestive enzymes (130). Furthermore, young broilers supplemented with 1 g/kg APS markedly enhanced BW and reduced FCR by enhancing activities of lipase, amylase and protease (131). In addition, 10 g/kg APS addition significantly enhanced BW, improved the intestinal morphology, enhanced VH and the ratio of VH/CD in jejunum, as well as reduced CD of the duodenum (132). In addition to the application of APS on livestock (pigs or broilers), APS has been reported to play potential role on the growth and development of fish (133). Sun and coauthors demonstrated that turbot (*Scophthalmus maximus L.*) supplemented with 150 mg/kg APS remarkably enhanced final body weight (FBW), specific growth rate (SGR), weight gain (WG), and ADFI by improving the activity of digestive enzymes (134). Consistently, crucian carps orally administrated with 100 mg/kg APS markedly enhanced body weight gain rate (BVGR), SGR and reduced the FCR (135). Additionally, 30 g/kg APS addition exerted beneficial effects on body protein composition, body weight gain, feed efficiency of white shrimp, and lipid metabolism (*Litopenaeus vannamei*) (136). Moreover, 0.01% APS in the diet of zebrafish significantly upregulated TJ protein 1b and Occludin1, improved intestinal permeability and promoted intestinal health. Furthermore, APS supplementation enhanced BW and reduced FCR (136).

Collectively, the improvement of APS on growth performance is attributed to intestinal villus morphology improvement, intestinal digestion and absorption as well as digestion enzymes activities improvement. Main functions of APS on animal growth performance were displayed in Table 2.

**Table 2:** Main functions of APS on animal’s growth performance.

Source	Application form	Experiment object	Main function	Reference
Astragalus polysaccharide purity 80%	Basal diet supplemented with 800 mg/kg	Weaned piglets	Enhanced the ADG and FCR	(125)
Astragalus polysaccharide contained 95% carbohydrate	Corn and soybean meal-based diet with 0.1%	Weaned piglets	Improved ADG and F/G ratio	(126)
Astragalus polysaccharide	*In ovo* injection of 2 mg/egg	Eggs	Enhanced the FI, BW, and FCR	(127)
*Astragalus kahericus* polysaccharide	4.5 mg *in ovo* injections	Cobb broiler chicks	Improved carcass percentage and FCR	(129)
*Astragalus membranaceus* polysaccharide	Dietary supplementation with 1,000 mg/kg APS	Juvenile broilers	Enhanced BW and reduced FCR	(135)
Astragalus polysaccharide	Basal diet supplemented with 10,000 mg/kg	Avein breeder cocks	Enhanced BW	(132)
Gamma-irradiated *Astragalus* polysaccharides purity 87.64%	Basal diet supplemented with 600 mg/kg	Ross-308 chicks	Enhanced ADG and reduced F/G ratio	(105)
Sulfated Astragalus polysaccharide purity 97%	Injected intramuscularly with 8 mg/kg of BW	Arbor acres broiler chicks	Enhanced BWG and reduced F/G ratio	(102)
Astragalus polysaccharide purity 70.23%	Basal diet supplemented with 300 mg/kg	Arbor acres broilers	Enhanced ADG and reduced F/G ratio	(74)
Astragalus polysaccharide	A concentrated solution (2 mg/mL) was prepared in 0.9% physiological saline	SPF Leghorn fertilized eggs	Enhanced the body weight at 1 day and final weight	(100)
Astragalus polysaccharide purity 50%	Basal diet supplemented with 150 mg/kg	*Scophthalmus maximus L.*	Enhanced FBW, SGR, WG, and FI	(134)
*Astragalus membranaceus* polysaccharide	A dose of 100 mg/kg with oral administration	Crucian carps	Improved the BVGR, SGR, and FCR	(135)
*Astragalus membranaceus* polysaccharide	Basal diet supplemented with 30,000 mg/kg	*Litopenaeus vannamei*	Improved the body protein level, body weight gain, and feed efficiency	(136)
Astragalus polysaccharide purity 60%	Basal diet supplemented with 0.01%	Zebrafish	Enhanced BW and reduced FCR	(137)

## 6 Conclusion

Astragalus polysaccharide is a natural bioactive component and possesses a variety of biological activities involved in anti-oxidation, anti-aging, anti-fibrosis, anti-tumor, antiviral and antibacterial, blood sugar reduction, blood lipid reduction, anti-fibrosis, and radiation protection effects. Furthermore, emerging evidence demonstrated that APS plays a potential role in intestinal epithelial barrier integrity maintenance, intestinal microbiota regulation, immune response, and redox homeostasis, which are critical for intestinal health, immune response, and animal’s growth performance. It can provide reference for further study on the effect of APS on intestinal barrier. In addition, it can be seen from the above numerous reports that Astragalus polysaccharide is widely used in the intestine, and there will be some breakthroughs in the future. On the one hand, origin, extraction, separation and purification methods contribute to the activities of APS, thus these processes are needed to be improved. On the other hand, the biological activities of APS are related to their chemical structure. APS is formed by galactose, glucose, mannose, rhamnose, xylose, arabinose, glucuronic acid, and galacturonic acid with condensation reaction, but accurate molecular structures of APS are largely unknown. Additionally, further understanding the regulatory role of APS in the interaction between microbiota and intestinal barrier are needed. In this context, analysis structure and further understanding the regulatory mechanical role of APS are crucial for the application of APS on intestinal health. APS might be a therapeutic intervention for intestinal disease. Therefore, APS will be a potential research object with broad prospects.

## Author contributions

HL: Data curation, Resources, Writing – original draft. ST: Resources, Writing – original draft. YaW: Resources, Writing – original draft. JZ: Resources, Writing – original draft. CY: Resources, Writing – original draft. YiW: Resources, Writing – original draft. NL: Funding acquisition, Supervision, Writing – original draft, Writing – review & editing. YQ: Funding acquisition, Resources, Writing – review & editing.

## Glossary

AFLD: Alcoholic fatty liver disease
APS: Astragalus polysaccharide
BPD: Bronchopulmonary dysplasia
CD: Crypt depth
CPM: Cyclophosphamide
DAO: Diamine oxidase
DSS: Dextran sulfate sodium
FCR: Feed conversion rate
FT-IR: Fourier transmission-infrared spectroscopy
GALT: Gut associated lymphoid tissue
GC: Gas chromatography
GLP-1: Glucagon-like peptide-1
HAPS: Honey-processed Astragalus polysaccharide
HPLC: High performance liquid chromatography
IBD: Inflammatory bowel disease
IBS: Irritable bowel syndrome
ICAM-1: Intercellular adhesion molecule 1
IFN-γ: Interferon gamma
Ig: Immunoglobulin
IL: Interleukin
ILF: Isolated lymphoid follicles
LPS: Lipopolysaccharide
MLN: Mesenteric lymph nodes
NDV: Newcastle disease vaccine
NF-κB: Nuclear factor-κB
NLRP: NoD-like receptor
NLRP3: NOD-like receptor thermal protein domain associated protein 3
NMR: Nuclear magnetic resonance spectroscopy
PAH: Pulmonary arterial hypertension
PPs: Peyer’s patches
ROS: Reactive oxygen species
SCFAs: Short-chain fatty acids
sIgA: Secretory immunoglobulin A
SOD: Superoxide dismutase
TCM: Traditional Chinese medicine
T-AOC: Total antioxidant capacity
TLR: Toll-like receptor
TJ: Tight junctions
VH: Villus height
VH/CD: Villus height/crypt depth
WG: Weight gain.
